# Genome-Wide Association Analysis Identified *BMPR1A* as a Novel Candidate Gene Affecting the Number of Thoracic Vertebrae in a Large White × Minzhu Intercross Pig Population

**DOI:** 10.3390/ani10112186

**Published:** 2020-11-22

**Authors:** Qian Liu, Jingwei Yue, Naiqi Niu, Xin Liu, Hua Yan, Fuping Zhao, Xinhua Hou, Hongmei Gao, Lijun Shi, Lixian Wang, Ligang Wang, Longchao Zhang

**Affiliations:** Institute of Animal Science, Chinese Academy of Agricultural Sciences, Beijing 100193, China; liuqian521818@163.com (Q.L.); qgww007@126.com (J.Y.); 18734450897@163.com (N.N.); firstliuxin@163.com (X.L.); zcyyh@126.com (H.Y.); zhaofuping@caas.cn (F.Z.); 7hxh73@163.com (X.H.); gaohongmei_123@126.com (H.G.); shilijun01@caas.cn (L.S.); ligwang@126.com (L.W.)

**Keywords:** *BMPR1A*, *FOS*, genome-wide association study, number of thoracic vertebrae, pig

## Abstract

**Simple Summary:**

The number of thoracic vertebrae (NTV) and number of vertebrae (NV) varies among pig breeds with a high correlation of about 0.8. It is important to discover variants associated with the NTV by considering the effect of the NV in pig. The results suggest that regulation variants on SSC7 might play crucial roles in the NTV and the *FOS* on SSC7 should be further studied as a critical candidate gene. In addition, *BMPR1A* was identified as a novel candidate gene affecting the NTV in pigs.

**Abstract:**

The number of vertebrae (NV), especially the number of thoracic vertebrae (NTV), varies among pig breeds. The NTV is controlled by vertebral segmentation and the number of somites during embryonic development. Although there is a high correlation between the NTV and NV, studies on a fixed NV have mainly considered the absolute numbers of thoracic vertebrae instead of vertebral segmentation. Therefore, this study aimed to discover variants associated with the NTV by considering the effect of the NV in pigs. The NTV and NV of 542 F2 individuals from a Large White × Minzhu pig crossbreed were recorded. All animals were genotyped for *VRTN* g.19034 A > C, *LTBP2* c.4481A > C, and 37 missense or splice variants previously reported in a 951-kb interval on SSC7 and 147 single nucleotide polymorphisms (SNPs) on SSC14. To identify NTV-associated SNPs, we firstly performed a genome-wide association study (GWAS) using the Q + K (population structure + kinship matrix) model in TASSEL. With the NV as a covariate, the obtained data were used to identify the SNPs with the most significant genome-wide association with the NTV by performing a GWAS on a PorcineSNP60K Genotyping BeadChip. Finally, a conditional GWAS was performed by fixing this SNP. The GWAS showed that 31 SNPs on SSC7 have significant genome-wide associations with the NTV. No missense or splice variants were found to be associated with the NTV significantly. A linkage disequilibrium analysis suggested the existence of quantitative trait loci (QTL) in a 479-Kb region on SSC7, which contained a critical candidate gene *FOS* for the NTV in pigs. Subsequently, a conditional GWAS was performed by fixing M1GA0010658, the most significant of these SNPs. Two SNPs in *BMPR1A* were found to have significant genome-wide associations and a significant dominant effect. The leading SNP, S14_87859370, accounted for 3.86% of the phenotypic variance. Our study uncovered that regulation variants in *FOS* on SSC7 and in *BMPR1A* on SSC14 might play important roles in controlling the NTV, and thus these genetic factors may be harnessed for increasing the NTV in pigs.

## 1. Introduction

The mammalian spine is composed of the following five types of morphologically different vertebrae: cervical, thoracic, lumbar, sacral, and caudal. In pigs, the number of cervical vertebrae is fixed at seven, as in almost all mammals [1]. Both the number of thoracic vertebrae (NTV), which is equal to the number of ribs (NR), and the number of lumbar vertebrae are variable in pigs. The term “number of vertebrae” (NV) is generally used for referring to the total number of thoracic and lumbar vertebrae [2]. The NTV and NV in pigs vary, ranging between 13 and 16, and between 19 and 22, respectively [3]. In Chinese pork markets, ribs are among the most valuable parts of the animal. In addition, the higher the NTV is, the longer the animal [4]. Hence, increasing the NTV has been an important goal in pig husbandry. Recently, molecular approaches have increasingly been sought to generate pigs with a desired NTV since it is difficult to characterize the NTV in live animals for phenotypic selection.

The NTV in pigs is a highly heritable trait (heritability from 0.24 to 0.78) and shows considerable variation across breeds [3,5]. Many studies have shown that the NTV is a polygenic trait influenced by many different quantitative trait loci (QTL) on *Sus scrofa* Chromosomes (SSCs) 1, 2, 4, 7, 11, 16, and 18 [6,7,8,9,10,11,12,13,14]. Genome-wide association studies (GWASs) in recent years have shown that the major genes controlling the NTV in pigs are concentrated on SSC7 [15,16,17]. It is known that the total number of vertebrae is determined during development by the number of somites, developing from the presomitic mesoderm (PSM) via the Notch, FGF, Wnt, and Retinoic acid pathways. Concurrently, the vertebral pattern, which refers to the division of cervical, thoracic, lumbar, sacral, and caudal vertebrae along the embryo anterior–posterior axis, is resolved according to the expression patterns of *Hox* genes [18]. The genetic correlation coefficient between the NTV and NV ranged from 0.79 to 0.97 in pigs [19]. These findings suggest that number of thoracic or lumbar vertebrae are controlled by both somite segmentation and vertebral patterning. Although we have previously discovered similar QTLs for the NTV [16,20] as in other studies [6,7,8,9,10,11,12,13,14], our previous view on the NTV as an independent trait [20] seems incomplete because of insufficient consideration of the effect of the NV. Hence, the aim of this study was to discover genome-wide association variants for the NTV in pigs by considering the effect of the NV.

## 2. Materials and Methods

### 2.1. Ethics Statement/Sample Description

All animals were sacrificed by electric shock in Beijing Fifth Meat Processing Factory according to the guidelines for experimental animals established by the Council of China. The experiments involving animals were approved by the Science Research Department of the Institute of Animal Science, Chinese Academy of Agricultural Sciences (CAAS) (Beijing, China) (No. IASCAAS-PG-39).

### 2.2. Phenotypic Data, Heritability, and Genetic Correlation between the NTV and NV

The phenotypic data included the NTV and NV of 542 individuals from the F2 progeny of 4 Large White pigs crossed with 16 Minzhu pigs. The F1 generation consisted of 9 boars and 46 sows which were mated to produce 542 F2 animals (65 L) in three parities. All these F2 individuals were slaughtered at 240 ± 7 d of age, and the phenotypes obtained from the carcasses were recorded. Genetic parameters pertaining to the NTV and NV were estimated using DMU software with pedigree-based linear models that are based on the following equation [21]:

*y* = *µ* + *a* + *e* (1)

where *y* is the NTV or NV of the animal, *µ* is the overall mean, *a* is the random additive genetic effect of the animal, and *e* is the random residual effect.

### 2.3. Genotyping for VRTN g.19034 A>C 37, VRTN g.20311_20312ins291, LTBP2 c.4481A>C, and Missense or Splice Variants on SSC7 and 147 SNPs on SSC1

All the animals were genotyped for “Vertebrae Development Associated” (*VRTN*) g.19034 A>C, g.20311_20312ins291 [18] and “Latent Transforming Growth Factor Beta-binding Protein 2” (*LTBP2*) c.4481A > C [15]. Through comparison with the *Sus scrofa* genetic variant database in Ensembl (http://asia.ensembl.org/Sus_scrofa/Location/Variant), 37 missense or splice variants in a previously reported 951-kb interval on SSC7 [16] were obtained, and the animals were then genotyped for these variants as well. The information about the above 39 mutations and the primers used for genotyping are displayed in Supplementary Table S1. Additionally, a total of 147 SNPs (single nucleotide polymorphisms) on SSC14 were genotyped for each individual. The primers used for these variants were designed using Primer3 (http://primer3.ut.ee/) (see Supplementary Table S2). Genomic DNA from each individual was extracted and used as the template for touchdown polymerase chain reaction (PCR). Touchdown PCR was carried out in 25-μL volumes containing 0.2 μM of each primer, 1.5 mM MgCl_2_, 0.2 mM of each deoxynucleotide triphosphate, 4 ng/μL genomic DNA, and 0.03 U/μL Taq DNA polymerase with 1× buffer (TaKaRa, Beijing, China) under the following conditions: 94 °C for 5 min; 5 cycles at 60 °C for 20 s and 72 °C for 45 s; 30 cycles at 52 °C for 20 s and 72 °C for 45 s; 72 °C for 10 min. The PCR products were resolved using 1.5% agarose-gel electrophoresis (Promega, Madison, WI, USA) and photographed under UV light. They were then purified and sequenced on an ABI 377 sequencer (ABI, Foster City, CA, USA).

### 2.4. Genome-Wide Association Study Based on the SNPs and Variants That Merged with Porcine SNP60K Genotyping BeadChip Assays

Population structure and cryptic relationships were considered to minimize false positives and increase statistical power. The kinship derived from the whole-genome SNPs was set as a random effect to control for family effects. The GWAS for the NTV was performed using the TASSEL 5.0 (Institute for Genomic Diversity, Cornell University, Ithaca, NY, USA) software [22] with the data from Porcine SNP60K BeadChip genotyping [20]. A sample call rate of >90%, an SNP call rate of >90%, a minor allele frequency of >5%, a maximum missing rate of <0.9, and only two alleles were used to evaluate data quality. A mixed linear model (Q + K) was applied in this GWAS. Principal component analysis (PCA) was performed. The kinship matrix was calculated using the default method (centered IBS (Identity by state)) [23] in TASSEL software. The “Q” matrix was determined using the PCA which was run on the genotype data set to account for the effects resulting from the population structure, and the kinship matrix (K) was calculated to replace pedigrees. The genotype at each SNP site and the first three PCAs were included as fixed effects, and a polygenic genetic effect was fitted as a random effect. The additive and dominant effects were estimated by the TASSEL software. The Bonferroni test whole-genome significance threshold was defined as 0.05/total SNPs (*p* = 1.10 × 10^−6^). The phenotypic variation explained by each SNP is calculated based on a formula for R^2^ using Tassel software as shown here (https://bitbucket.org/tasseladmin/tassel-5-source/wiki/UserManual/MLM/ MLM).

(2) $$R^{2}=\frac{{\left({\hat{Y}}_{full}-{\hat{Y}}_{reduced}\right)}^{T}V^{-1}\left({\hat{Y}}_{full}-{\hat{Y}}_{reduced}\right)}{{\left(Y-\bar{Y}\right)}^{T}V^{-1}\left(Y-\bar{Y}\right)}$$

Since SNP M1GA0010658 showed the maximum association with the NTV, a conditional GWAS was re-run with this SNP fixed to detect novel associated variants. We calculated the distance between each significant genome-wide SNP and the annotated genes upstream and downstream of the SNP by using the Ensembl database (http://asia.ensembl.org). The nearest gene to the SNP was considered as a candidate gene for NTV.

### 2.5. Identification of the Effect of S14_87859370 on the NTV in Large White and Songliao Black Pigs

A total of 214 Large White (LW) pigs from VICA group Co., Ltd. (Huludao, China), and 129 Songliao Black (SLB) pigs from the Jilin Academy of Agricultural Sciences were used in this study. The NTV was recorded after slaughter. Genotypic effects were analyzed using a one-way ANOVA through the GLM procedure of the SAS software package version 8.2 (SAS Institute, Inc., Cary, NC, USA) with genotype as a fixed effect. Duncan’s multiple-range tests were used for assessing the significance of the differences among the means of different genotypes.

### 2.6. Linkage Disequilibrium Analysis

Linkage disequilibrium analysis was performed on the chromosomal region which contained all the SNPs significantly associated with the NTV on SSC7 and SSC14, respectively. The genotypes of all SNPs for 542 individuals and pedigrees were used to detect haplotype blocks. The visualized haplotype blocks were detected using the HAPLOVIEW V3.31 program [21] with default parameters.

## 3. Results

### 3.1. Phenotypes and Genetic Parameters Pertaining to the NTV and NV

The NTV and NV phenotypes observed are summarized in Table 1. The NTV was found to be 14, 15, and 16 in 193, 308, and 41 individuals, respectively. Interestingly, there were no individuals with 14 thoracic vertebrae and 22 vertebrae or with 16 thoracic vertebrae and 20 vertebrae. Using DMU software, the heritability values of the NTV and NV were estimated to be 0.59 and 0.54, respectively. The genetic correlation between the two traits reached 0.77.

### 3.2. GWAS with the NTV by Using the Merged SNP Data from 542 F2 Animals Revealed Significant Variants

**Figure 1:**
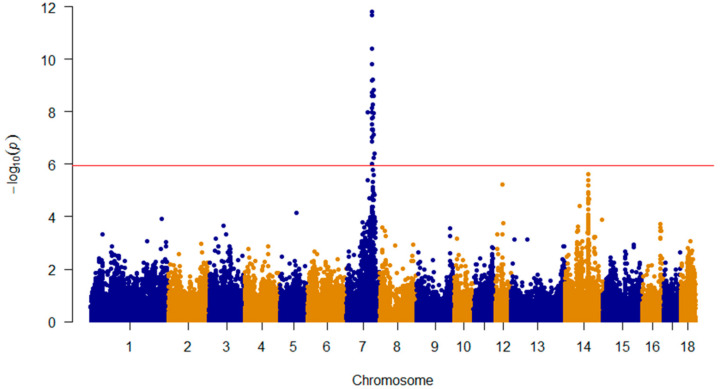
The Manhattan plot for genome-wide association studies for the NTV. The genome-wide association study (GWAS) was run on the NV treated as a covariate based on the 147 SNPs on SSC17 and 39 variants on SSC7 that merged with porcine SNP60K genotyping BeadChip assays. The red horizontal line indicated the Bonferroni significance threshold (1.10 × 10^−6^).

Using the merged SNP data obtained from the 542 F2 individuals, a GWAS revealed a strong association peak (QTL) on SSC7 (Figure 1). The Q-Q plot (Quantile-Quantile plot) is shown in Supplementary Figure S1A. A total of 31 SNPs in a 25.76-Mb region (from 80.26 to 106.03 Mb) on SSC7 showed significant genome-wide associations with the NTV (Table 2). The SNPs most significantly associated with the NTV corresponded to the variations of M1GA0010658 in “Placental Growth Factor” (*PGF*), and these variations could explain 7.80% of the phenotypic variance. All 31 SNPs showed significant additive effects with no dominant effect. This region contained a previously reported 951-kb interval, which comprised the reported candidates *VRTN* and *LTBP2* for the NTV. In addition, 37 non-synonymous variants were detected during the whole-genome sequencing, and each animal was genotyped for these variants. For *VRTN* g.20311_20312ins291, no polymorphism was detected in this population. To evaluate the effects of these variants, *LTBP2* c.4481 A > C, and *VRTN* g.19034 A > C, we identified them through a GWAS. The results suggested that the effects of these variants and two genes on the NTV were not significantly associated with the trait at the genome-wide level (Table 3). However, there were 1 and 5 SNPs on SSC 12 and 14, respectively, that had chromosome-wide associations with the NTV (Supplementary Table S3). The candidate genes were “Carbonic Anhydrase 10” (*CA10*) on SSC12 and *BMPR1A* on SSC14.

### 3.3. Linkage Disequilibrium Analysis Suggested Candidate Genes Are Contained in a 479-Kb Region on SSC7

**Figure 2:**
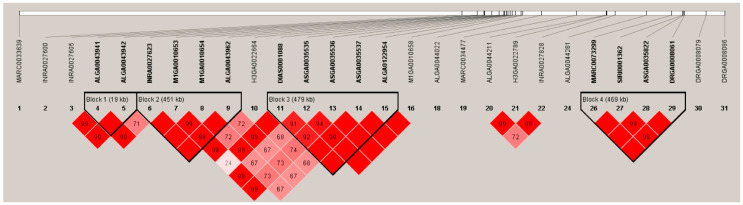
Haplotypes on a 25.76-Mb region on SSC7 containing all SNPs associated with the NTV significantly. The analysis was obtained using the HAPLOVIEW 3.31 program. A total of four blocks were identified and marked with solid lines.

Within the 25.76-Mb region on SSC7, the linkage disequilibrium analysis detected four haplotype blocks as ALGA0043941-ALGA0043942 for 19 kb, ALGA0043941-ALGA0043962 for 451 kb, DIAS0001088-ALGA0122954 for 479 kb, and MARC0073299-DRGA0008061 for 469 kb (Figure 2). Since the 479-Kb block is the closest to the most significant SNP M1GA0010658, it could be regarded as the most important region harboring the candidate genes for the NTV. The 479-Kb region contained nine candidate genes: ribosomal protein S6 kinase like 1 (*RPS6KL1*), eukaryotic translation initiation factor 2B subunit beta (*EIF2B2*), mutL homolog 3 (*MLH3*), acylphosphatase 1 (*ACYP1*), NIMA related kinase 9 (*NEK9*), zinc finger C2HC-type containing 1C (*ZC2HC1C*), transmembrane p24 trafficking protein 10 (*TMED10*), Fos proto-oncogene, AP-1 transcription factor subunit (*FOS*), and Jun dimerization protein 2 (*JDP2*).

### 3.4. Conditional GWAS and Haplotype Block Analysis Revealed BMPR1A as a Novel Candidate Gene Affecting the NTV

**Figure 3:**
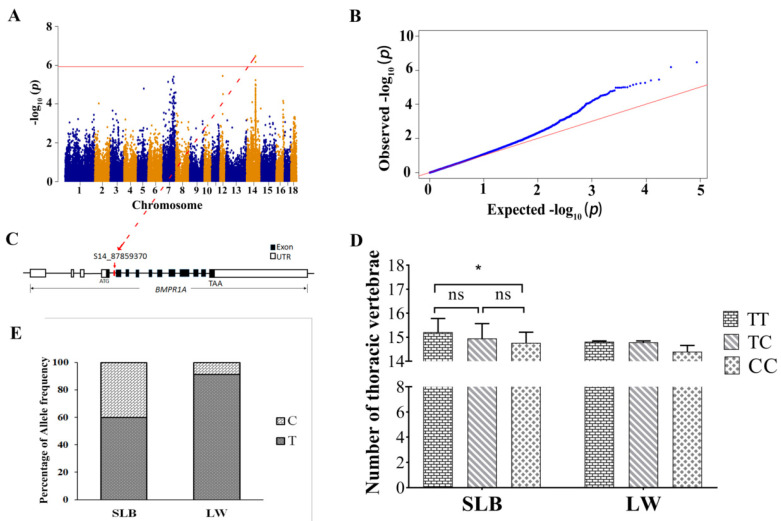
Identification of *BMPR1A* using conditional GWAS of the NTV. (**A**) Manhattan plot displaying the GWAS results of the NTV. The red horizontal line indicated the Bonferroni significance threshold (1.10 × 10^−^^6^). Dots represent SNPs and the S14_87859370 is highlighted at the top dot. (**B**) The Q-Q plots obtained from the conditional GWAS for the NTV. (**C**) Red arrow represents the most significant SNP signal of the NTV. The S14_87859370 in the intron 4 of BMPR1A is marked in red. (**D**) The difference analysis among three genotypes of S14_87859370 for NTV in Songliao Black (SLB) and Large White (LW) pig populations. * indicates *p* < 0.05. (**E**) Allele frequencies of S14_87859370 in SLB and LW pig populations.

A conditional GWAS was performed by fixing the strongest significant SNP, M1GA0010658, on SSC7. Only the SNPs S14_87859370 and S14_87859377, located on SSC14, were determined to be significantly associated with the NTV (Figure 3A,B). The two SNPs were found to have significant dominant effects with no additive effects. The Q-Q plot is shown in Supplementary Figure S1B. Both were located in BMPR1A. The leading SNP was S14_87859370, which resided in intron 4 of BMPR1A (Figure 3C) and accounted for 3.86% of the phenotypic variance in the NTV (Table 4).

To estimate the effect of S14_87859370 on the NTV, we genotyped pure lines of Songliao Black and Large White pigs for this SNP (Table 5). The Songliao Black pigs with the TT genotype had more NTV than those with the CC genotype (*p* < 0.05) (Figure 3D). Although the Large White pigs with the TT genotype had more thoracic vertebrae than those with the TC or CC genotype, the difference did not reach statistical significance since there were only two individuals with the CC genotype (Figure 3E). Nevertheless, in both lines, the frequency of the T allele was higher than that of the C allele (Figure 3E).

## 4. Discussion

In this study, we did not observe any animals with an NTV of 14 or 16 in the populations with an NV of 22 or 20, respectively. The heritability of the NTV and NV was similar to that in previous reports [5]. The high genetic correlation (0.77) found in this study between the two traits indicates that the NTV and NV are closely related. Therefore, genetic analyses regarding the NTV should fully consider the effect of the NV. With the NTV considered an independent variable, only one QTL, located on SSC7, has been discovered [16,17]. However, when we considered the effect of the NV, our GWAS for the NTV detected 31 SNPs with genome-wide associations and located within a 22.93-Mb region (from 80.26 to 103.19 Mb) on SSC7, one chromosome-wide association SNP at 28.24 Mb on SSC12, and five chromosome-wide association SNPs within a 104.12-kb region (from 87.80 to 87.90 Mb) on SSC14. The 22.93-Mb region on SSC7 also included a previously reported 951-kb fragment consisting of important candidate genes—namely *VRTN*, *LTBP2*, and “Visual System Homeobox 2” (*VSX2*) [13,15,16,17,24,25,26]. Among these candidates, *VRTN* g.19034 A > C [17] and *LTBP2* c.4481A > C [15] have been regarded as good candidate causal mutations. However, the association of either of them with the NTV did not reach genome-wide significance in our F2 population. Furthermore, a total of 37 non-synonymous variants addressed also displayed no significant association with the NTV in our GWAS.

On SSC7, a 479-Kb region including nine annotated genes was identified as a critical fragment influencing the NTV. Among these nine genes, *FOS*, also known as AP-1, is an important member of the Notch signaling pathway [27] and could be treated as the most important candidate gene for the NTV. The activation of *FOS* could inhibit the expression of Notch-1 [28], which is a critical gene for somite development [29]. That homozygous *fos* −/− mice displaying growth-retarded, develop osteopetrosis with deficiencies in bone remodeling and tooth eruption [30] suggested that this gene played an important role during bone development. Similar to a previous report [24], *FOS* was also regarded as a critical candidate gene for the porcine NTV in this work.

Surprisingly, when the effect of SSC7 was fixed, two variants on SSC14 showed significant genome-wide associations with the NTV. In addition, different from the SNPs on SSC7, we detected dominant effects at the two SNPs. To our knowledge, this is the first report with a dominant effect on NTV. These results suggested that the NTV was a complex trait with additive and dominant effects. Both of them were located in *BMPR1A*, which was selected as a good candidate due to its essential role in BMP (Bone morphogenetic protein) signaling [31]. *BMPR1A*, also known as ALK-3 and Brk-1, encodes a type I “Transforming Growth Factor-β” (TGF-β) family receptor for the BMP-2 and BMP-4 signaling pathways [32]. In mice, BMP-2 is strongly expressed in the mid-somite region (anterior trunk of the embryo), and BMP-4 is robustly expressed in dorsal somites along the entire embryonic axis at E10.5 [33]. BMPs are secreted proteins that interact with cell-surface receptors to cause bone differentiation [34] and are believed to play various important roles during vertebrate embryogenesis [32]. The activation of the BMP signaling pathways requires the binding of BMPs to a hetero-oligomeric complex composed of type 1 and type 2 BMPRs. Three type 1 receptors (BMPR1a/Alk3, BMPR1b/Alk6, and ACVR1A/Alk2) and three type 2 receptors (BMPR2, ACTR2A, and ACTR2b) mediate most of the effects of BMPs [35]. Following the binding of BMPs to BMPR1/2 receptor heterodimers, receptor-regulated Smads (R-Smads) are activated. Subsequently, these activated R-Smads form hetero-oligomeric complexes with a common mediator Smad (co-Smad) and translocate into the nucleus, where they regulate the transcription of target genes [36,37]. Smads can modulate the transcriptional activities of Hox proteins, which act as general downstream DNA-binding proteins in the BMP/Smad signaling cascade [38]. *Hox* genes are key regulators of morphogenesis along the axial skeleton [39]. Vertebral patterning depends on ordered patterns of *Hox* gene expression as a mechanism for generating a combinatorial code that specifies the unique identities of the segments and their derivatives [40]. BMP signaling regulates vertebral specification when differentiating somites [41], and its perturbation in somitogenesis results in vertebral and rib malformations in the axial skeleton [42]. The discovery that mice lacking *BMPR1A* die by E9.5 due to defects in mesoderm formation [32] has led to the recognition of this protein as an essential factor for somitogenesis and vertebral development. Therefore, *BMPR1A* expression may control vertebral patterning via classical BMP-Smad-Hox signaling. These results indicate that S14_87859370, located in *BMPR1A*, is a potential binding site for a transcriptional regulator of the BMP pathway, and this observation may have important implications for further studies on the regulation of the NTV in pigs.

## 5. Conclusions

Our results suggest that regulation variants on SSC7 might play crucial roles affecting NTV. The *FOS* on SSC7 should be further studied as a critical candidate gene. On SSC14, to our knowledge, this is the first report showing that a natural variant in *BMPR1A* significantly contributes to the NTV in pigs. The identified variations provide valuable molecular information that may be harnessed to increase NTV in pigs.

## Figures and Tables

**Table 1:** The distribution of the number of thoracic vertebrae in different groups with different numbers of thoracolumbar vertebrae.

Traits		NTV		
		14	15	16
NV	20	146	88	0
	21	47	209	19
	22	0	11	22

NTV, number of thoracic vertebrae. NV, number of vertebrae.

**Table 2:** Genome-wide association studies identified some associated variations for the NTV on SSC7 ^1^.

Marker	Chr ^2^	Position ^3^	Rs ^4^	*p*-Value	Add_*p* ^5^	Dom_*p* ^6^	Nearest Gene	Var (%) ^7^
MARC0033639	7	80264245	rs80875505	1.06 × 10^−8^	2.00 × 10^−12^	1.02 × 10^−2^	*SLC12A6*	5.18
INRA0027600	7	96435072	rs333341186	1.33 × 10^−7^	2.38 × 10^−12^	9.66 × 10^−3^	*RBM25*	4.44
INRA0027605	7	96994146	rs337851273	9.12 × 10^−8^	4.69 × 10^−12^	3.78 × 10^−2^	*ENSSSCG00000002348*	4.55
ALGA0043941	7	97247184	rs81396045	2.56 × 10^−9^	2.05 × 10^−11^	7.49 × 10^−2^	*FAM161B*	5.59
ALGA0043942	7	97266221	rs80856304	4.84 × 10^−8^	7.41 × 10^−11^	6.97 × 10^−3^	*COQ6*	4.74
INRA0027623	7	97521999	rs321816080	1.03 × 10^−8^	3.53 × 10^−10^	1.14 × 10^−2^	*VSX2*	5.18
M1GA0010653	7	97795697	rs81396078	1.72 × 10^−8^	4.32 × 10^−10^	8.41 × 10^−4^	*LTBP2*	5.037
M1GA0010654	7	97954258	rs80864705	1.87 × 10^−9^	2.37 × 10^−10^	8.79 × 10^−2^	*FCF1*	5.68
ALGA0043962	7	97973860	rs80929215	1.87 × 10^−9^	2.37 × 10^−10^	8.79 × 10^−2^	*YLPM1*	5.68
H3GA0022664	7	98066911	rs80813473	3.11 × 10^−8^	8.24 × 10^−10^	1.09 × 10^−3^	*PROX2*	4.863
DIAS0001088	7	98116120	rs336641062	6.52 × 10^−10^	3.30 × 10^−10^	1.11 × 10^−1^	*RPS6KL1*	5.99
ASGA0035535	7	98186259	rs80963494	1.49 × 10^−10^	4.32 × 10^−9^	1.45 × 10^−2^	*EIF2B2*	7.01
ASGA0035536	7	98264173	rs80846252	3.92 × 10^−11^	9.72 × 10^−10^	3.34 × 10^−2^	*ACYP1*	6.91
ASGA0035537	7	98374939	rs80854726	7.21 × 10^−9^	4.42 × 10^−9^	1.97 × 10^−1^	*TMED10*	5.30
ALGA0122954	7	98595714	rs81317665	2.03 × 10^−12^	2.17 × 10^−9^	7.21 × 10^−4^	*JDP2*	7.74
M1GA0010658	7	98648325	rs80804788	1.55 × 10^−12^	9.66 × 10^−9^	2.23 × 10^−2^	*PGF*	7.80
ALGA0108658	7	98648325	rs81336593	9.67 × 10^−7^	4.26 × 10^−9^	1.22 × 10^−2^	*JDP2*	3.88
ALGA0044022	7	99337831	rs80919617	5.78 × 10^−10^	2.22 × 10^−9^	4.40 × 10^−3^	*GPATCH2L*	6.04
MARC0034477	7	100621452	rs80954820	4.91 × 10^−8^	2.16 × 10^−6^	2.02 × 10^−1^	*SPTLC2*	4.74
ALGA0044211	7	101703098	rs80950372	5.03 × 10^−8^	1.01 × 10^−8^	3.80 × 10^−3^	*NRXN3*	4.72
H3GA0022789	7	101728012	rs80933409	5.52 × 10^−9^	1.03 × 10^−8^	6.25 × 10^−2^	*NRXN3*	5.37
INRA0027828	7	102031355	rs323989598	1.60 × 10^−8^	7.11 × 10^−9^	2.05 × 10^−2^	*NA*	5.06
ASGA0035786	7	103002983	rs80802872	2.46 × 10^−9^	1.71 × 10^−8^	6.72 × 10^−3^	*DIO2*	5.63
ALGA0044281	7	103164950	rs80808662	1.17 × 10^−8^	1.15 × 10^−8^	8.36 × 10^−3^	*NA*	5.15
H3GA0022821	7	103189827	rs80970878	1.45 × 10^−9^	1.17 × 10^−8^	8.56 × 10^−3^	*DIO2*	5.76
MARC0073299	7	104087830	rs80786139	1.15 × 10^−8^	1.17 × 10^−8^	8.56 × 10^−3^	*STON2*	5.15
SIRI0001362	7	104480447	rs320949387	7.60 × 10^−8^	7.80 × 10^−7^	7.64 × 10^−2^	*ENSSSCG00000049060*	4.60
ASGA0035822	7	104525604	rs80936448	7.68 × 10^−8^	4.22 × 10^−7^	1.40 × 10^−1^	*ENSSSCG00000049060*	4.60
DRGA0008061	7	104557781	rs80813073	7.68 × 10^−8^	9.73 × 10^−6^	4.92 × 10^−1^	*ENSSSCG00000049060*	4.60
DRGA0008079	7	105341213	rs80962000	5.53 × 10^−7^	8.99 × 10^−8^	1.66 × 10^−2^	*ENSSSCG00000047467*	4.03
DRGA0008086	7	106025579	rs81295294	3.95 × 10^−7^	3.22 × 10^−7^	1.99 × 10^−1^	*ENSSSCG00000045920*	4.13

^1^ NTV, number of thoracic vertebrae. ^2^ Chromosome ^3^ Data from *Sus scrofa* Build 11.1. ^4^ rs, reference SNP. ^5^ add_p, *p*-value for additive effect. ^6^ dom_p, *p*-value for dominant effect. ^7^ Var (%), phenotypic variation explained by the SNP.

**Table 3:** Association of 37 missense or splice region variants, *VRTN* g.19034 A > C, and *LTBP2* c.4481 A > C on SSC7.

Marker	Chr ^1^	Position ^2^	Rs ^3^	Var% ^4^	*p*-Value
S7_97537758	7	97537758	rs336742966	0.67	8.99 × 10^−2^
*VRTN* g.19034 A > C	7	97614602	rs709317845	2.99	2.41 × 10^−5^
S7_97622681	7	97622681	rs787326242	1.31	1.01 × 10^−2^
S7_97623045	7	97623045	rs1108261998	1.42	6.57 × 10^−3^
S7_97662010	7	97662010	rs696186042	0.52	1.58 × 10^−1^
S7_97662082	7	97662082	rs345827854	2.02	9.68 × 10^−4^
S7_97662535	7	97662535	rs332888554	1.19	1.51 × 10^−2^
S7_97750084	7	97750084	rs322330509	2.91	3.91 × 10^−5^
*LTBP2* c.4481 A > C	7	97751432	rs322260921	2.95	2.83 × 10^−5^
S7_97765472	7	97765472	rs339379718	1.6	3.58 × 10^−3^
S7_97771260	7	97771260	rs337082599	1.69	2.92 × 10^−3^
S7_97775923	7	97775923	rs335686067	0.24	4.45 × 10^−1^
S7_97777490	7	97777490	rs331228271	0.49	1.88 × 10^−1^
S7_97895559	7	97895559	rs341911129	1.49	5.30 × 10^−3^
S7_97899571	7	97899571	rs325918746	1.21	1.24 × 10^−2^
S7_97901617	7	97901617	rs322374710	2.63	8.55 × 10^−5^
S7_97901619	7	97901619	rs331788516	2.63	8.55 × 10^−5^
S7_98073512	7	98073512	rs80930259	2.78	5.24 × 10^−5^
S7_98073927	7	98073927	rs329005836	0.97	3.36 × 10^−2^
S7_98074140	7	98074140	rs339766519	1.5	4.63 × 10^−3^
S7_98074438	7	98074438	rs322346679	2.73	8.41 × 10^−5^
S7_98116877	7	98116877	rs344681928	0.18	5.21 × 10^−1^
S7_98130124	7	98130124	rs323664885	0.03	9.07 × 10^−1^
S7_98203930	7	98203930	rs787271115	0.36	2.77 × 10^−1^
S7_98219169	7	98219169	rs323701300	0.1	7.04 × 10^−1^
S7_98219967	7	98219967	rs344167352	0.29	3.52 × 10^−1^
S7_98242037	7	98242037	rs338693270	0.11	6.67 × 10^−1^
S7_98242461	7	98242461	rs694346166	0.56	1.29 × 10^−1^
S7_98242725	7	98242725	rs340407061	0.92	3.68 × 10^−2^
S7_98243724	7	98243724	rs713439416	1.08	2.03 × 10^−2^
S7_98244079	7	98244079	rs333141847	0.19	5.08 × 10^−1^
S7_98266495	7	98266495	rs329334983	1.98	1.57 × 10^−4^
S7_98266534	7	98266534	rs324580288	1.76	3.68 × 10^−4^
S7_98266749	7	98266749	rs342214814	1.58	3.35 × 10^−3^
S7_98266963	7	98266963	rs693150674	1.72	1.98 × 10^−3^
S7_98279107	7	98279107	rs323090151	1.84	1.36 × 10^−3^
S7_98300295	7	98300295	rs341533265	0.96	3.05 × 10^−2^
S7_98451235	7	98451235	rs319445329	0.28	3.68 × 10^−1^
S7_98451601	7	98451601	rs80846787	0.43	2.25 × 10^−1^

^1^ Chromosome. ^2^ Data from *Sus scrofa* Build 11.1. ^3^ Reference SNP. ^4^ Phenotypic variation explained by the significant SNPs.

**Table 4:** Conditional genome-wide association study for the NTV ^1^.

Marker	Chr ^2^	Position ^3^	*p*-Value	Add_*p* ^4^	Dom_*p* ^5^	Nearest Gene	Position in Gene	Var (%) ^6^
S14_87859370	14	87859370	3.40 × 10^−7^	2.38 × 10^−3^	6.71 × 10^−8^	*BMPR1A*	Intron	3.86
S14_87859377	14	87859377	6.79 × 10^−7^	2.28 × 10^−3^	1.51 × 10^−7^	*BMPR1A*	Intron	3.65

^1^ NTV, number of thoracic vertebrae. ^2^ Chromosome. ^3^ Data from *Sus scrofa* Build 11.1. ^4^ *p*-value for additive effect. ^5^ *p*-value for dominant effect. ^6^ Phenotypic variation explained by the significant SNPs.

**Table 5:** The association of S14_87859370 with the NTV in Songliao Black and Large White pigs ^1^.

Breed	Number	Genotype	Number of Thoracic Vertebrae ^2^
Songliao Black	39	TT	15.21 ± 0.57 ^a^
	77	TC	14.95 ± 0.62 ^ab^
	13	CC	14.77 ± 0.44 ^b^
Large White	179	TT	14.82 ± 0.03
	33	TC	14.79 ± 0.06
	2	CC	14.40 ± 0.26

^1^ NTV, number of thoracic vertebrae ^2^ Completely different superscript letters indicate significant differences (*p* < 0.05).

## Supplementary Materials

The following are available online at https://www.mdpi.com/2076-2615/10/11/2186/s1, Table S1: The information and primers for *VRTN* g.19034 A > C, *LTBP2* c.4481A > C, and 37 missense or splice region variants in a reported 951-kb interval on SSC7, Table S2: The primers for 147 SNPs on SSC14, Table S3: Genome-wide association studies identified some chromosome-wide significant associated variations for the NTV, Figure S1: The Q-Q plots obtained from genome-wide association studies for the NTV.
