# Supplementary material for: Genome-Wide Association Analysis Identified BMPR1A as a Novel Candidate Gene Affecting the Number of Thoracic Vertebrae in a Large White × Minzhu Intercross Pig Population
Source: Animals (Basel). 2020 Nov 22;10(11):2186. doi: 10.3390/ani10112186 (PMC7700692; doi:10.3390/ani10112186)
Supplement: Supplementary file 1 [file animals-10-02186-s001.zip › Supplementary File/Figure S1.docx]

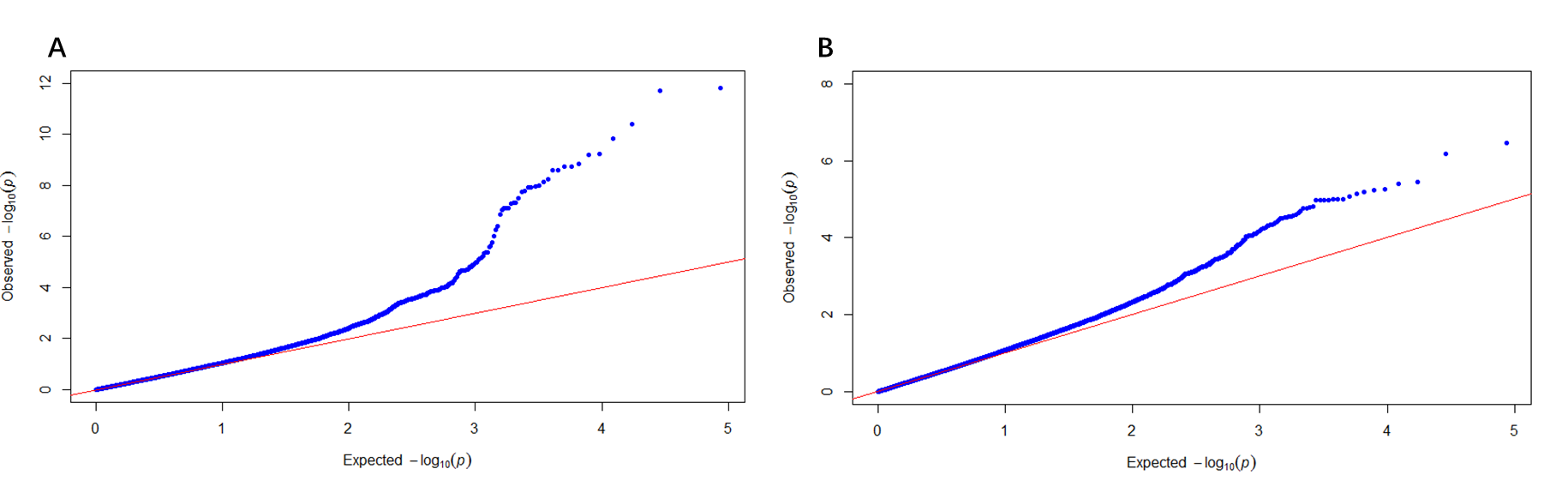


**Figure S1** The Q-Q plots obtained from genome-wide association studies for NTV. A Q-Q plot for TNV by GWAS with NV as covariate. B Q-Q plot for TNV by GWAS with NV as covariate and fixing the strongest significant SNP M1GA0010658 on SSC7.
