# Supplementary material for: Genome-Wide Association Analysis Identified BMPR1A as a Novel Candidate Gene Affecting the Number of Thoracic Vertebrae in a Large White × Minzhu Intercross Pig Population
Source: Animals (Basel). 2020 Nov 22;10(11):2186. doi: 10.3390/ani10112186 (PMC7700692; doi:10.3390/ani10112186)
Supplement: Supplementary file 1 [file animals-10-02186-s001.zip › Supplementary File/Table S2.docx]

**Table S2** The primers for 147 SNPs on SSC14

| SNP^1^ | Primer up | Primer down | Amplification size (bp) |
| --- | --- | --- | --- |
| S14_81854473 | TGCATAGCCTTTTCAGACTGG | GGCTGAAAACATGTGCACAC | 246 |
| S14_81940137 | CCACCGAACTTCCATTTAC | CGTCTCCTTTGGCTATTACA | 549 |
| S14_81940144 |  |  |  |
| S14_82682311 | TTGCTGATACTTTCCACT | ACAGACTTACCTTTCCTC | 590 |
| S14_83325697 | GCCATTCCCAGACATAAA | CTAAGCCCAGCTCGACTA | 785 |
| S14_85597043 | TTCTTAACTCTTTGCCTGAA | AATGATTCCTTGGTCCCT | 435 |
| S14_86454978 | ATCCCTCAGAATGTTAGT | ATGTTTCAGTCCTCCAAT | 630 |
| S14_86868589 | AAGATAGTTCCCTGATGTG | AATAGGTCCTAGCTGTGC | 344 |
| S14_86868605 |  |  |  |
| S14_86868640 |  |  |  |
| S14_86931003 | AGCAGTTGCCCAATAAAC | TTGAGACCAGGGTGAAAT | 472 |
| S14_86997417 | TATCATTGGGATCATTTGG | GGGTTATCATCTAAGGCAC | 598 |
| S14_87023930 | TGCTTGCCTGACAGAACA | TGACAGCCCAGATGGAGA | 612 |
| S14_87031402 | TGGCAGGTCCCTTATTCC | TCTTGCTGGCTCTGTCCC | 602 |
| S14_87087511 | GCCTCTAGCTTGGACTCTG | GGCTTCCTGGTGGTATGT | 448 |
| S14_87142542 | AGCATAGGCGGCATTTGT | GGGTGTCAGAGGCGAGTT | 391 |
| S14_87187561 | CTGCCACAAGGGATAAAC | CTGCCTCTGTGACCTACAC | 492 |
| S14_87195435 | ATAAGAAATGTGCGAGAC | CTGATTACTGGAGCCTAG | 494 |
| S14_87236025 | GTGAGCCCAGCCCTACAT | TCTCCTGCCTGGCTTCTC | 572 |
| S14_87254294 | CCCAAGAAGGGAACAGAT | TGTCTAGCAGGCAGACCA | 662 |
| S14_87254535 |  |  |  |
| S14_87298658 | CCACAACAACTTGGGATG | AGGAGGCAAAGACTCGTA | 704 |
| S14_87298787 |  |  |  |
| S14_87337958 | AGGACAGAGTGGACGGAATA | AGTCGAACTGGAGCGAGA | 503 |
| S14_87647167 | GCCTGAATCTGCGGCTAT | TTCGGGCTTCTGGACTTT | 662 |
| S14_87647302 | ACACCCGCAGAGGACATT | CCCAGTCACTCAGCACCT | 711 |
| S14_87647344 |  |  |  |
| S14_87647417 |  |  |  |
| S14_87647438 |  |  |  |
| S14_87651752 | GGAGTCTACCGCCCTTCT | CTCTGCTACCCAATCTTGC | 454 |
| S14_87652436 | CTTCCCAGGTGCCTATGT | GGGTCACGGCTAACTTCA | 549 |
| S14_87654027 | CTTGGTCGTGCTGGTTTG | GCCATGCTGGGATTCTATT | 709 |
| S14_87654295 | GCCTCAGCTTGGAGGGAT | GGCTAATGGGAGTGGACAA | 562 |
| S14_87734239 | GTGGCTACTGAACCCTGAG | GATACCTGCTCTTCTCCC | 758 |
| S14_87735171 | CATTCTACTGCAACGCTGAG | GGCATAGGCTGGCAACTA | 508 |
| S14_87735404 | TATGCCAAATACCCAGAA | AATCCAACCAGGAACAAT | 326 |
| S14_87738289 | TGAAAGAATGGAGGATGT | GCTGAGATAACTGGGAAG | 531 |
| S14_87745648 | ATTCCACCTCTAAACATTG | TCTCCCATCATACACCAG | 449 |
| S14_87750754 | GCCACTTGTTGACAGATA | GTAGCAAGGGATCAATCT | 555 |
| S14_87751992 | TACAGACAGTGCTATGGTGCTT | TCGCTCAATGGGTTACGG | 542 |
| S14_87752181 | GAAGGCATGGAGATTTGA | TGTGGTGTAGGTCGCAGA | 422 |
| S14_87752717 | GGGATCAAACCTGCAACC | GGAGGGCAAGAAGGACAT | 686 |
| S14_87756484 | TTTGGAGTTTGAGTTGAT | TATTTAGACCCTGAGTGG | 618 |
| S14_87756842 | CCGCAAAGAGTAGGTAGG | ATCATGGCGCAGCAGAAA | 389 |
| S14_87757188 | TATGGAGGTTCCCAGGAC | CATGAGGATGCGGGTTTG | 493 |
| S14_87772313 | TTAGGGAAACACCCACAG | AAGGAAGCCATCAACAAA | 544 |
| S14_87772378 | TTAGGGAAACACCCACAG | AAGGAAGCCATCAACAAA | 544 |
| S14_87780448 | TCTAATCTTTGGGGAACA | TGTGGATGTGATGTAGGC | 503 |
| S14_87781900 | TCTAAGCAGTCTTCCCATAA | TTGCCTTGTCTACATCAGC | 653 |
| S14_87782620 | GGAAAGGAGGAAGAAGAG | CCTAATGCCTAATGTGATG | 529 |
| S14_87782667 | AGTCCAAGATCAAGGTGCTG | CATGAGGATGTGGGTTCG | 405 |
| S14_87782963 | TGGCAGAAGGAGCAAGGA | CAAAGGGTCAGAGGGAGG | 669 |
| S14_87785675 | CCGTTTCTGAGGGTTTAA | GGAAGTGAGATGGAGGGA | 644 |
| S14_87785696 | CTGTGGGATGCAGAAGTT | GGAAGTGAGATGGAGGGA | 361 |
| S14_87785844 | GGATTGGACCTGTGCTAC | TTAAGAGCCCAGATGAAA | 423 |
| S14_87786531 | TAACCCACTGAGCAAGGC | CATAGCAACAGGGCATCT | 608 |
| S14_87786575 | TAACTACTGAGCCACGACG | CATAGCAACAGGGCATCT | 547 |
| S14_87787625 | TTTCTCCACATCATTTGGTA | GAAGATTCCCTCCTCACA | 612 |
| S14_87787654 | TTTCTCCACATCATTTGGTA | GAAGATTCCCTCCTCACA | 612 |
| S14_87789500 | TTCAAAGGCCACAGTTTA | CAAGGGAAGGTGAGGTAG | 644 |
| S14_87789627 | TTCAAAGGCCACAGTTTA | AAGGGAAGGTGAGGTAGA | 643 |
| S14_87793612 | TCCTTCCTCTGCCTATCT | GAGTTGAGTAAACCGACA | 479 |
| S14_87794413 | CTGTGGCTGGAGTTCTAC | GCCTCATACAGTGGGTTA | 679 |
| S14_87794610 | CCCCTAGAGTTAGCTGGTC | GTTCAATCCCTAGCCTCA | 461 |
| S14_87794714 | TTAATCCTACACCCAGTA | CTAAGTGCCATTTATCCC | 425 |
| S14_87796361 | TGGTTTATCTTGGTGAAT | AAGGAAAAGGAATAGGAA | 571 |
| S14_87798759 | TGCCTGTCTTGGGGAAAC | ACTGAAAGGGTGGGGATT | 438 |
| S14_87799252 | CCCCACCCTTTCAGTTAT | AACGCCATTTGCATCAAC | 519 |
| S14_87803294 | CCTTGTAGGAGTTCCCAGTA | ATTTCAACCCAGGCAGTT | 519 |
| S14_87803352 | TTGCTGTGGCTGTGGTAT | AACCTCTAGCCTGGGAAC | 710 |
| S14_87803851 | GGTTCCCAGGCTAGAGGT | TTTGGTCACTCCCACAGC | 403 |
| S14_87807118 | AATGTTGCTTGCTGGTAG | CAGGAAAGGGTAATGGAC | 551 |
| S14_87807646 | CTGTAGTCCATTACCCTTTC | ATCTTTCCTCCATCAGCA | 730 |
| S14_87808156 | TGCTGATGGAGGAAAGAT | CATGAGGACGCAGGTTCA | 360 |
| S14_87808192 | TGCTGATGGAGGAAAGAT | CATGAGGACGCAGGTTCA | 360 |
| S14_87818253 | TCTAGGAGTTCCCGTTGT | GAAGTTTAGCGAGTCATTTT | 442 |
| S14_87824329 | AGTTCCCATCGTGGCTCA | ACCGTGGCAGGCTAACAA | 534 |
| S14_87824785 | TTTCCCTCTGCATGTTGC | AATGTTGGTGGAGCCTTT | 427 |
| S14_87824797 | TTTCCCTCTGCATGTTGC | AATGTTGGTGGAGCCTTT | 427 |
| S14_87827663 | TACATTTCCTACCCTTACT | TTGGTTACCGTTATAGTC | 706 |
| S14_87832232 | CGGGAACTCCAAAGAAAT | TGTGGTGTAGGTCGCAGA | 455 |
| S14_87833420 | TGAGGTGGGCTTGTATTG | GAAGTTCCCTGGTTGTCC | 504 |
| S14_87835947 | CCTTCCCTCCCTCCATTT | GAGCAGCACCACAACAGC | 474 |
| S14_87844202 | CGTGGATAAAGGTAGAGG | AACCATTGCTTAGGGACT | 596 |
| S14_87850550 | GGAGTGGTTGCCAAATAA | CATGAGGTTGCAGGTTCA | 608 |
| S14_87850639 | TGCTGGGACAAATAAAGG | CATGAGGTTGCAGGTTCA | 547 |
| S14_87852463 | AGTATCGGGTTTGAGGAT | GGAATATGAGGCTGAAGAC | 605 |
| S14_87852705 | CCTCACTCCCTTACCACC | CCTAGCCTGTGAACCTCC | 493 |
| S14_87858783 | GTGTAGCTGCTGATGGAC | AAGTGTTTACCCTTTCTTG | 569 |
| S14_87859370 | CACAAGAAAGGGTAAACA | TCTATTATGCCACTCCAC | 684 |
| S14_87859377 | CACAAGAAAGGGTAAACA | TCTATTATGCCACTCCAC | 684 |
| S14_87862861 | AGATGACCAGGGAGAAAC | AAATCCGACTAGGAACCA | 521 |
| S14_87868945 | TGCCCTTATCCTTACCAA | GTGACAACACCAGACCCT | 464 |
| S14_87869022 | CTGCCTCCTTGACTACCT | GTGACAACACCAGACCCT | 383 |
| S14_87873365 | TACAGCTCCAATTCAACC | TTCCCATATCTTATTTCTCC | 468 |
| S14_87874814 | GGGAGGCACAGGTTCAGT | ATAAAGCACCCCAGTTGA | 436 |
| S14_87877094 | GTAAGATAGAGCCACAGG | GCTTTATGTTATCACCCT | 518 |
| S14_87877191 | GAGAAGACACTTCCCTCAA | CCAGATCCTTAACCCACT | 681 |
| S14_87877208 | ACTGATTGGCTGGGAGAA | CCACTTATGAAGGGTTTT | 591 |
| S14_87879714 | TGTGGTGTTGGTGGTTAC | TCCTCATCCCTCTTATTT | 565 |
| S14_87881335 | ACAGATGGTTCGGCAAGT | AAGAGGCAAGGAAGGGTC | 686 |
| S14_87881837 | CACATGGCTTTCTTTCCT | GCCTTGCTTAGTGGGTTA | 537 |
| S14_87890902 | TGGCTTCTGTCTGTCATT | AGCACTAAGGCTGAACTG | 727 |
| S14_87891158 | TTAAGTTGGCCCAAGATC | ATTCCGTTGAAAGCACAG | 450 |
| S14_87891873 | TAGACATTGAAAGAAGGGTT | GAGACCAGGGTTGGAACT | 538 |
| S14_87900179 | GTTTCCCTTGGTTGACTC | TTTGTTCCTCCTGTGGTT | 374 |
| S14_87900479 | TTAGCCTGCTGAGCCACC | ACCTTCCGCAAAGACAAT | 452 |
| S14_87907339 | TGAAACGGCACTGTCCTA | CTGGCTCCAAGTTGTTCTC | 679 |
| S14_87915033 | CTCTTACCTCTTCCCATCCG | GCCTCTTGCCAAGTGTTATCT | 708 |
| S14_87917248 | CACGGTGCTGGTGAGTAGCT | CAGGCGAACTGTGACTGGA | 484 |
| S14_87931468 | GTGTATCCAGATGCTCCCTTAT | GGACCTGCTCCACGAAAA | 640 |
| S14_87931531 | GGACTTCAGGCGTCAGAAAC | GGGACCTGCTCCACGAAAA | 395 |
| S14_87931548 | TGTGACAGCCGCACCCTA | GGGACCTGCTCCACGAAAA | 303 |
| S14_87931620 | CCCACCCAGTTGTCTCGT | ATTACGGACAGAGGGAATAGC | 462 |
| S14_87931630 | CCCACCCAGTTGTCTCGT | ATTACGGACAGAGGGAATAGC | 462 |
| S14_87942664 | TTTTGGTGGTCTTCCTTT | CTACTCACGCCTCGGTTT | 516 |
| S14_88027874 | TCTGCTTATTTCGCTATG | CTCCAGTTTGACCCTATT | 470 |
| S14_88039117 | TCCCAACAAGTAAGAAAGT | ACAAATACCAATCGAGGC | 653 |
| S14_88048419 | ACAGCTCATGGCAACACC | ACCACGGAAATTCCCTCT | 561 |
| S14_88064399 | TTATCTTCCCTGTGCTCC | TGTGGCTCTGGTTACTGC | 363 |
| S14_88070997 | AGGCTTTCTTACTATTACTC | CATTGAGGTCCTTTGTTG | 476 |
| S14_88071016 | GTCATGGCTCAGTGGTAA | CAGAATGCGTTGTTCTATT | 466 |
| S14_88074044 | AAGCAGGAAGGAATAACC | GTAGCCACCGACCTACAC | 589 |
| S14_88078944 | GAACATGCACAAATGCCAAAT | TAACCACTGCGCCACGAC | 616 |
| S14_88080317 | CCATTTATTCCCAGGTGT | TGGAGGTTTCCAGGTTAG | 530 |
| S14_88100458 | AGAACTCAGATTGGGACT | AAGGGATGGAGTAGAATAG | 725 |
| S14_88117460 | GCACCACAAAGGGAACAC | CCCAGCACCATTTACCAA | 403 |
| S14_88117519 | AAATCACAACAGGCTTCC | TATTACCTTGCCCTTCAC | 434 |
| S14_88117752 | GTAAATGGTGCTGGGATA | AGTGCCTGTTCATGGATC | 422 |
| S14_88121052 | TTTGTCCTTCTGCCTTCC | GATCTGAGCCGTGTCTGC | 502 |
| S14_88126927 | GGGGTGCTTAGATGGGTT | AGGAGCTGCTTCCTTGTTT | 429 |
| S14_88126946 | GGGGTGCTTAGATGGGTT | AGGAGCTGCTTCCTTGTTT | 429 |
| S14_88298676 | CAAAGGAGAAATAAGGGAC | TTGCTGAGGGAGGAGTGT | 596 |
| S14_88360725 | TCCCAGCCCACTGACTTA | AAAGAGCAGGGTGAAAGC | 480 |
| S14_88377849 | TCAACAGATAAACGGATAAAGG | GCAAAGGCAGCAGAAGTG | 610 |
| S14_88431832 | CCAGACCTCACCGTTCCA | CCCGCTCAATTCCTCACTA | 417 |
| S14_88442999 | GACTAAATGTTTGAGGCTAT | ATTTGGCTGTTTCTGTGA | 603 |
| S14_88443024 | GACTAAATGTTTGAGGCTAT | ATTTGGCTGTTTCTGTGA | 603 |
| S14_88472541 | AAAATGCACAAGGGAGTA | TGAACCCAGATGAAAGGA | 473 |
| S14_88581090 | CTCCTGCTATGGGTTGAA | GAGGTGGACTTTATGACTGAC | 381 |
| S14_89145127 | CCCTGGGATCTGAGCATT | TCTGGGTATAGCGGTTGG | 438 |
| S14_87806064 | TTCCATACCTAGCAGCAT | AATCCCAACAGCCAAGAC | 422 |
| S14_87888320 | AAAATGGTGCTTTCTGTG | CTATAATTGGCAAATCTTGG | 362 |
| S14_87890042 | GATCTACTCATTCTAGGGTT | AAGACTTTTGGATTTCAA | 559 |
| S14_87890127 |  |  |  |
| S14_87891058 | TTTTGTATTGCCTTTCCA | CATGTGATGCTGCCCTAG | 534 |
| S14_87891377 | AGCCTTAGTGCTGTTATG | AACCCTTCTTTCAATGTC | 331 |

^1^The SNPs were named using its positions on Chromosome.
