# Supplementary material for: Genome-Wide Association Analysis Identified BMPR1A as a Novel Candidate Gene Affecting the Number of Thoracic Vertebrae in a Large White × Minzhu Intercross Pig Population
Source: Animals (Basel). 2020 Nov 22;10(11):2186. doi: 10.3390/ani10112186 (PMC7700692; doi:10.3390/ani10112186)
Supplement: Supplementary file 1 [file animals-10-02186-s001.zip › Supplementary File/Table S3.docx]

**Table S3** Genome-wide association studies identified some chromosome-wide significant associated variations for NTV^1^

| Marker | Chr^2^ | | Pos^3^ | P-value | | Nearest gene | Var(%)^4^ |
| --- | --- | --- | --- | --- | --- | --- | --- |
| CASI0009146 | 12 | 28244736 | | 5.96E-06 | CA10 | | 3.36 |
| S14_87796361 | 14 | 87796361 | | 1.32E-05 | BMPR1A | | 3.19 |
| S14_87803851 | 14 | 87803851 | | 1.15E-05 | BMPR1A | | 3.33 |
| S14_87859370 | 14 | 87859370 | | 2.33E-06 | BMPR1A | | 3.67 |
| S14_87859377 | 14 | 87859377 | | 4.18E-06 | BMPR1A | | 3.48 |
| S14_87900479 | 14 | 87900479 | | 6.61E-06 | BMPR1A | | 3.52 |

^1^NTV, number of thoracic vertebrae

^2^Chromosome

^3^Data from *Sus scrofa* Build 11.1

^4^Var(%), phenotypic variation explained by the SNP
