# Supplementary material for: Genome-Wide Association Analysis Identified BMPR1A as a Novel Candidate Gene Affecting the Number of Thoracic Vertebrae in a Large White × Minzhu Intercross Pig Population
Source: Animals (Basel). 2020 Nov 22;10(11):2186. doi: 10.3390/ani10112186 (PMC7700692; doi:10.3390/ani10112186)
Supplement: Supplementary file 1 [file animals-10-02186-s001.zip › Supplementary File/Table S1.docx]

**Table S1 The information and primers for *VRTN* g.19034 A>C, *LTBP2* c.4481A>C, and 37 missense or splice region variants in a reported 951-kb interval on SSC7**

| Variant | Allele | Chr | Position | rs number | Mutation type | Gene | Primer UP (5’ to 3’) | Primer Down (5’ to 3’) | Amplification size (bp) |
| --- | --- | --- | --- | --- | --- | --- | --- | --- | --- |
| S7_97537758 | C/T | 7 | 97537758 | rs336742966 | splice region variant | *VSX2* | TTGACTGTCTGGCTTTGC | CAATAGTCTTGTCCCTCCAC | 323 |
| *VRTN* g.20311_20312ins291 | In/del | 7 | 97616179 | - | intron indel | *VRTN* | AGGGGCAGATGAGGAGAATG | CTTGAGGTTGGAAGGGAGGA | 380 |
| *VRTN* g.19034 A>C | A/C | 7 | 97614602 | rs709317845 | intron variant | *VRTN* | CGGGAATCTGAGCTAGGTCA | GGTTTCATATCTGTGGTGGACC | 236 |
| S7_97622681 | C/T | 7 | 97622681 | rs787326242 | missense variant | *VRTN* | CTATCGCTGCTTCAAACGC | AGGGTGACAGGCTTGGGT | 356 |
| S7_97623045 | C/T | 7 | 97623045 | rs1108261998 | missense variant | *VRTN* | CTACCCAAGCCTGTCACCC | CATGTCCATCACCAGCACC | 343 |
| S7_97662010 | G/C | 7 | 97662010 | rs696186042 | missense variant | *SYNDIG1L* | CTGGGCTCCTTGACCTTG | CCCGGATCAGTGTACATCTG | 362 |
| S7_97662082 | T/C | 7 | 97662082 | rs345827854 | missense variant | *SYNDIG1L* | CCCCAGGAGGTAGGAATAG | CGAAGCAAGTTAGGTACAGAGT | 306 |
| S7_97662535 | A/G | 7 | 97662535 | rs332888554 | splice region variant | *SYNDIG1L* | AGGGTTACCGAGCATCAG | TATCCCGCTTGAAATGTG | 283 |
| S7_97750084 | G/A | 7 | 97750084 | rs322330509 | missense variant | *LTBP2* | CGGGAATTTGGTGGTGTC | GCCGGGCTATGAGTATGG | 358 |
| *LTBP2* c.4481A>C | A/C | 7 | 97751432 | rs322260921 | missense variant | *LTBP2* | GTCCTCACCCTCACACTTGT | CAAGACAGGCTGCAAACCC | 242 |
| S7_97765472 | G/C | 7 | 97765472 | rs339379718 | missense variant | *LTBP2* | TGGCATCAAGGGATTAGC | CATCGGGTGAGGAGACAG | 378 |
| S7_97771260 | G/T | 7 | 97771260 | rs337082599 | splice region variant | *LTBP2* | CTGTCCTCCAGCCCTTCC | GGTGCCTTGGGGTTTTAT | 375 |
| S7_97775923 | T/C | 7 | 97775923 | rs335686067 | missense variant | *LTBP2* | CATCTTTCCCCGTGTCCT | GAGACTCAGCTCGCTCTGC | 273 |
| S7_97777490 | G/A | 7 | 97777490 | rs331228271 | splice region variant | *LTBP2* | GGGTTGGGTGAAGGAGTTG | GTGTTGCAGCGGACAAGG | 308 |
| S7_97895559 | G/A | 7 | 97895559 | rs341911129 | missense variant | *AREL1* | AGAGGTCCTCTTGATGGAT | GCTTGGCTTTCTTGTTGA | 351 |
| S7_97899571 | C/T | 7 | 97899571 | rs325918746 | splice region variant | *AREL1* | TTGTAAGCACTCACCTCT | TCCACATCTTGTCCCTCT | 327 |
| S7_97901617 | T/C | 7 | 97901617 | rs322374710 | splice region variant | *AREL1* | GTCCCAACCAACCACATT | GAAACCGATGACCCAGAG | 261 |
| S7_97901619 | T/C | 7 | 97901619 | rs331788516 | splice region variant | *AREL1* |  |  |  |
| S7_98073512 | G/A | 7 | 98073512 | rs80930259 | splice region variant | *PROX2* | GTTCTTTGTTCCCACCTCC | ACTGCCCATCATTCCCTC | 175 |
| S7_98073927 | A/G | 7 | 98073927 | rs329005836 | missense variant | *PROX2* | CGTGGGCAGGCACAGTCA | GGGGCAATATCCCAGGCT | 316 |
| S7_98074140 | A/G | 7 | 98074140 | rs339766519 | missense variant | *PROX2* | AAGGCAGCCGGAGAAAGT | TGAGGGTAAAGCAGATGAATGG | 355 |
| S7_98074438 | T/C | 7 | 98074438 | rs322346679 | missense variant | *PROX2* | ACCCTGGAGGTGGTCACTG | GGAGCGGAAGAGGAAGCA | 286 |
| S7_98116877 | G/A | 7 | 98116877 | rs344681928 | missense variant | *RPS6KL1* | AAGCAGATAACCCCTGACTTT | GACCTCAATCCCCAGAACC | 163 |
| S7_98130124 | C/A | 7 | 98130124 | rs323664885 | missense variant | *RPS6KL1* | CGCCTCGTAGTCCTCACTG | TGGGTTTAACTTAATGCCTCTG | 265 |
| S7_98203930 | C/T | 7 | 98203930 | rs787271115 | missense variant | *EIF2B2* | TCTCCCACGCCAGAGTTAGT | GTTGCTCCAGCGGTAGTCC | 273 |
| S7_98219169 | T/G | 7 | 98219169 | rs323701300 | missense variant | *EIF2B2* | GTGGGCTGAAAGAAAGCA | AACGGTAGAGGAAGGTTAGT | 328 |
| S7_98219967 | G/A | 7 | 98219967 | rs344167352 | missense variant | *EIF2B2* | CTTGTTAAGGGACCTGTTT | ACACTCCGCATCTCAGTC | 314 |
| S7_98242037 | T/G | 7 | 98242037 | rs338693270 | splice region variant | *EIF2B2, MLH3* | TGCCATACATACACTTACA | AAATGAAGACTCCAACCC | 386 |
| S7_98242461 | T/C | 7 | 98242461 | rs694346166 | missense variant | *EIF2B2, MLH3* | AAGTTCCACTTTGGTCAG | AGTCAGGTTCCAGTAGGC | 354 |
| S7_98242725 | A/C | 7 | 98242725 | rs340407061 | missense variant | *EIF2B2, MLH3* | CCTACTGGAACCTGACTG | CACTACCTTGAAACATAA | 301 |
| S7_98243724 | G/A | 7 | 98243724 | rs713439416 | missense variant | *EIF2B2, MLH3* | TTCTTTCCTCTTCCTTTC | AAATCTTGCTTGGAACTT | 283 |
| S7_98244079 | C/T | 7 | 98244079 | rs333141847 | missense variant | *EIF2B2, MLH3* | ACTCTGAGCAAGCACTAT | TATTTAGCACCACTCTTC | 294 |
| S7_98266495 | C/T | 7 | 98266495 | rs329334983 | missense variant | *ZC2HC1C* | ACTCTTCCCAGCGGTCTT | CATCAGTGCCAGCCTCAC | 339 |
| S7_98266534 | A/G | 7 | 98266534 | rs324580288 | missense variant | *ZC2HC1C* | GGGGCACCTGAGGAATAA | AACCAAAACTGCTGTATGAG | 380 |
| S7_98266749 | G/A | 7 | 98266749 | rs342214814 | missense variant | *ZC2HC1C* | GGACCGAGCATACCCACT | TTCCTCCTCTGTCTTCTTCA | 325 |
| S7_98266963 | A/C | 7 | 98266963 | rs693150674 | missense variant | *ZC2HC1C* | GGCAAACCCCAGCAGGAT | CCGTCCCCAAGTTTCATC | 319 |
| S7_98279107 | C/T | 7 | 98279107 | rs323090151 | missense variant | *NEK9* | CTTCAAAGGTGGAAATCA | CTAGTCTCCTGAGCCTGTAT | 296 |
| S7_98300295 | A/C | 7 | 98300295 | rs341533265 | missense variant | *NEK9* | TGGGAATGAGTGGACAGA | GACAGCGTTCCTTATTGG | 278 |
| S7_98451235 | C/A | 7 | 98451235 | rs319445329 | missense variant | *FOS* | GTTACTGAATGTTGCCCTCT | GAGCTGCCAGGATGAACT | 368 |
| S7_98451601 | A/G | 7 | 98451601 | rs80846787 | missense variant | *FOS* | GCCTGCAAGATCCCTGATG | ACAGAGCGGGCTGTCTCA | 260 |
